# Injectable heat-sensitive nanocomposite hydrogel for regulating gene expression in the treatment of alcohol-induced osteonecrosis of the femoral head

**DOI:** 10.1063/5.0130711

**Published:** 2023-01-18

**Authors:** Zherui Fu, Yi Lai, Yaping Zhuang, Feng Lin

**Affiliations:** 1 Department of Emergency, The First People's Hospital of Xiaoshan District, Xiaoshan Affiliated Hospital of Wenzhou Medical University, Hangzhou, Zhejiang, China; 2 Department of Orthopedics, Shanghai Key Laboratory for Prevention and Treatment of Bone and Joint Diseases, Shanghai Institute of Traumatology and Orthopedics, Ruijin Hospital, Shanghai Jiao Tong University School of Medicine, 197 Ruijin 2nd Road, Shanghai 200025, People's Republic of China.; 3 Department of Orthopedics, The First People's Hospital of Xiaoshan District, Xiaoshan Affiliated Hospital of Wenzhou Medical University, Hangzhou, Zhejiang, China

## Abstract

For repairing lesions, it is important to recover physiological and cellular activities. Gene therapy can restore these activities by regulating the expression of genes in lesion cells; however, in chronic diseases, such as alcohol-induced osteonecrosis of the femoral head (ONFH), gene therapy has failed to provide long-term effects. In this study, we developed a heat-sensitive nanocomposite hydrogel system with a secondary nanostructure that can regulate gene expression and achieve long-term gene regulation in lesion cells. This nanocomposite hydrogel exists in a liquid state at 25 °C and is injectable. Once injected into the body, the hydrogel can undergo solidification induced by body heat, thereby gaining the ability to be retained in the body for a prolonged time period. With the gradual degradation of the hydrogel *in vivo*, the internal secondary nanostructures are continuously released. These nanoparticles carry plasmids and siRNA into lesion stem cells to promote the expression of B-cell lymphoma 2 (inhibiting the apoptosis of stem cells) and inhibit the secretion of peroxisome proliferators-activated receptors γ (PPARγ, inhibiting the adipogenic differentiation of stem cells). Finally, the physiological activity of the stem cells in the ONFH area was restored and ONFH repair was promoted. *In vivo* experiments demonstrated that this nanocomposite hydrogel can be indwelled for a long time, thereby providing long-term treatment effects. As a result, bone reconstruction occurs in the ONFH area, thus enabling the treatment of alcohol-induced ONFH. Our nanocomposite hydrogel provides a novel treatment option for alcohol-related diseases and may serve as a useful biomaterial for other gene therapy applications.

## INTRODUCTION

Recovering physiological and cellular activities is essential for lesion repair. When diseased cells in the lesion resume their normal physiological activities, the extracellular matrix can be effectively reconstructed and the original structure and function of the lesion can be restored.^1–3^ Gene therapy has proven to be an effective method of regulating cellular activity.^4^ Nanoscale biomaterial carriers have been widely used to develop biomaterials that can encapsulate genes due to their injectability and good tissue permeability. Through encapsulation into functional nanoparticles, a gene fragment can maintain its activity in the human body for an extended period of time. Moreover, gene fragments can be enriched in a lesion via local injection with a syringe, thereby avoiding any side effects resulting from systemic administration.^5–7^ For example, Bedingfield *et al.*^8^ alleviated cartilage damage in patients with traumatic osteoarthritis by delivering siRNA via nanoparticles. Pang *et al.*^9^ used nanoparticles loaded with delta-5-desaturase siRNA to inhibit the formation of lung tumors, and Lee *et al.*^10^ used RNA nanoparticles to inhibit the oncogenic expression of miRNA-21 to treat glioblastoma. Nanoparticles can greatly improve the efficacy of gene therapy and reduce its side effects. However, due to their nanoscale size, they are readily absorbed and cleared by the blood and lymphatic capillaries, resulting in a short treatment duration and the need for repeated administration, which ultimately increases the treatment burden and pain in patients.^11^ To circumvent these limitations, we propose an innovative nanoparticle–hydrogel composite delivery system. By loading nanoparticles on a hydrogel, it can resist the removal of the nanoparticles by biological tissues and provide a stable microenvironment for the nanoparticles, which is more conducive to the maintenance of gene expression activity.

Osteonecrosis of the femoral head (ONFH) is a clinically common condition that poses a serious threat to human health and is characterized by a high disability rate.^12,13^ Nontraumatic ONFH is more common in young and middle-aged individuals, and heavy drinking is one of its main causes. Without effective treatment, functional damage of the joint and loss of mobility may occur, thereby increasing the rate of disability and seriously affecting the quality of life of these patients.^14–16^ Therefore, it is important to develop therapeutics for the prevention and treatment of alcohol-induced ONFH. Previous studies have demonstrated alcohol-induced apoptosis of mesenchymal stem cells (MSCs) in the femoral head, which results in the generation of necrotic areas in the femoral head and microfractures in the subchondral bone, an important pathophysiological mechanism for the induction of ONFH.^17^ Moreover, alcohol induces lipid accumulation in the femoral head, which increases the pressure inside the bone marrow cavity and blocks blood circulation, thereby aggravating ONFH.^18^ Therefore, inhibiting apoptosis and adipogenic differentiation of stem cells using gene therapy may be an effective strategy for treating ONFH. Previous studies have indicated that an increase in the expression of B-cell lymphoma 2 (Bcl-2) inhibits stem cell apoptosis in patients with ONFH. CircRNA-3503 can enhance the expression of peroxisome proliferator-activated receptor-gamma coactivator 1 alpha (PGC-1α) by acting as an RNA sponge to adsorb micRNA-181c-3p. High expression of PGC-1α can induce the expression of the downstream target Bcl-2, thereby inhibiting stem cell apoptosis.^19^ Meanwhile, the secretion of peroxisome proliferators-activated receptors γ (PPARγ) is an important mechanism leading to the adipogenic differentiation of stem cells.^20^ Therefore, the adipogenic differentiation of stem cells in patients with ONFH may be significantly inhibited through the prevention of PPARγ synthesis via siRNA.

Thus, it is necessary to construct an injectable nanocomposite hydrogel system for delivering gene fragments that can regulate Bcl-2 and PPARγ expression in the femoral head and achieve a sustained release as an effective treatment for patients with ONFH. At present, most hydrogels are crosslinked and form a solid state *in vitro* (e.g., crosslinked by UV irradiation); however, such hydrogels can only be surgically implanted into the body, greatly limiting their use, particularly for the treatment of ONFH. The most mature clinical nonsurgical technique for the treatment of ONFH is the maintenance of the femoral head through injection. Based on this clinical technique, hydrogels need to be developed that can be crosslinked and solidified *in vivo* using body heat. This eliminates the need to inject additional chemical cross-linking agents into the body, thereby greatly reducing the potential side effects. Therefore, we constructed a nanocomposite hydrogel using a thermal crosslinked hydrogel. This hydrogel is in the liquid state outside of the body and can be injected into the femoral head using a syringe. Subsequently, body heat causes a transition from liquid to solid state inside the body. These solid hydrogels provide a stable microenvironment for nanoparticles, thus preventing them from being catabolized and removed by the body. This achieves a slow-release, long-term treatment for femoral head necrosis.

**Scheme 1.**
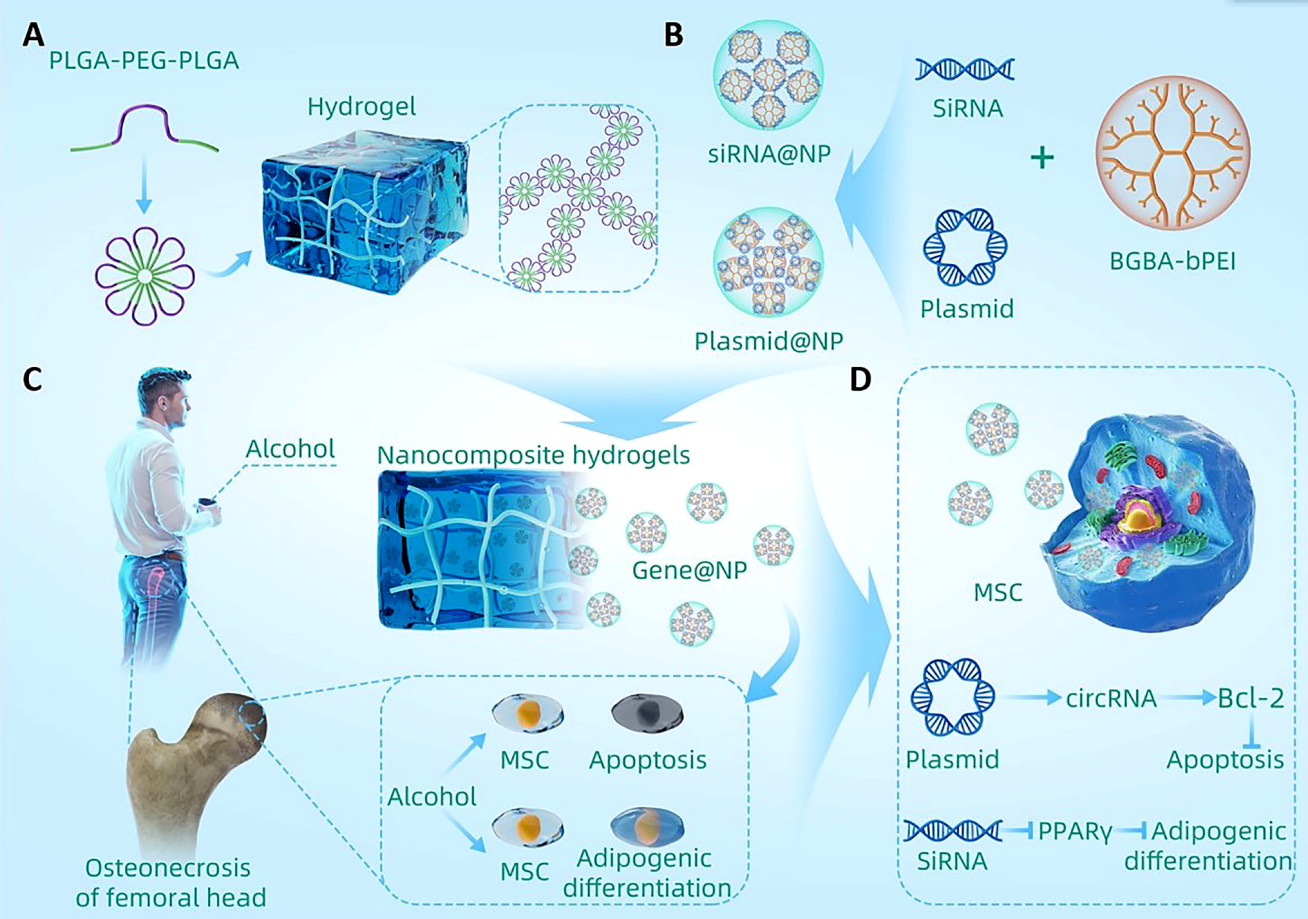
Schematic diagram of the nanocomposite hydrogel for the treatment of alcohol-induced ONFH. (a) Schematic diagram of the heat-sensitive hydrogel synthesis. (b) Schematic diagram of the gene-loaded nanoparticle synthesis. (c) Pathophysiological mechanism of chronic alcohol consumption that leads to ONFH. (d) Nanocomposite hydrogels inhibit the apoptosis and adipogenic differentiation of MSCs, thus treating ONFH.

In this study, we constructed a novel, heat-sensitive nanocomposite hydrogel system with a secondary nanostructure that can regulate gene expression over long term in focal cells. The nanocomposite hydrogel exists in a liquid state at room temperature and is injectable. Once injected into patients with ONFH resulting from long-term alcoholism, the hydrogel undergoes solidification induced by body heat, thereby gaining the ability to be retained in the body for a prolonged time period. With the gradual degradation of the nanocomposite hydrogel *in vivo*, the internal secondary nanostructures are continuously released. Nanoparticles can deliver regulatory genes into local stem cells to regulate their gene expression. In particular, when plasmid-carrying nanoparticles enter stem cells, they, especially designed geoparticles, use the transcription system of the target stem cells to transcribe and generate circRNA-3503. This circRNA enhances the expression of Bcl-2 and effectively inhibits alcohol-induced apoptosis of stem cells by acting as an RNA sponge to adsorb micRNA-181c-3p. These siRNA-carrying nanoparticles can inhibit the expression of PPARγ in stem cells through the release of siRNA, thereby inhibiting the alcohol-induced adipogenic differentiation of stem cells. Thus, the normal physiological function of the stem cells can be restored (Scheme 1). Our *in vivo* experiments using a rat model of alcohol-induced ONFH demonstrate that the nanocomposite hydrogel system can effectively inhibit the apoptosis and adipogenic differentiation of MSCs in the femoral head to restore the normal physiological function of local MSCs and effectively repair femoral head lesions. Through the detection of several key proteins during femoral head repair, we demonstrate that the nanocomposite hydrogel system repairs alcohol-induced ONFH-associated lesions. Our findings suggest that this nanocomposite hydrogel system is a promising biomaterial carrier for gene therapy and represents an alternative treatment for alcohol-related diseases.

## RESULTS AND DISCUSSION

### Characterization of gene-loaded nanoparticles

Gene-loaded nanoparticles were developed based on the self-assembly properties of dendritic macromolecules and genes. Subsequently, a series of experiments were conducted to characterize the morphology, diameter, and charge of the nanoparticles. Finally, it was verified that the nanoparticles were successfully loaded with the gene of interest by analyzing various chemical elements contained in dendritic macromolecules and genes.

**FIG. 1.**
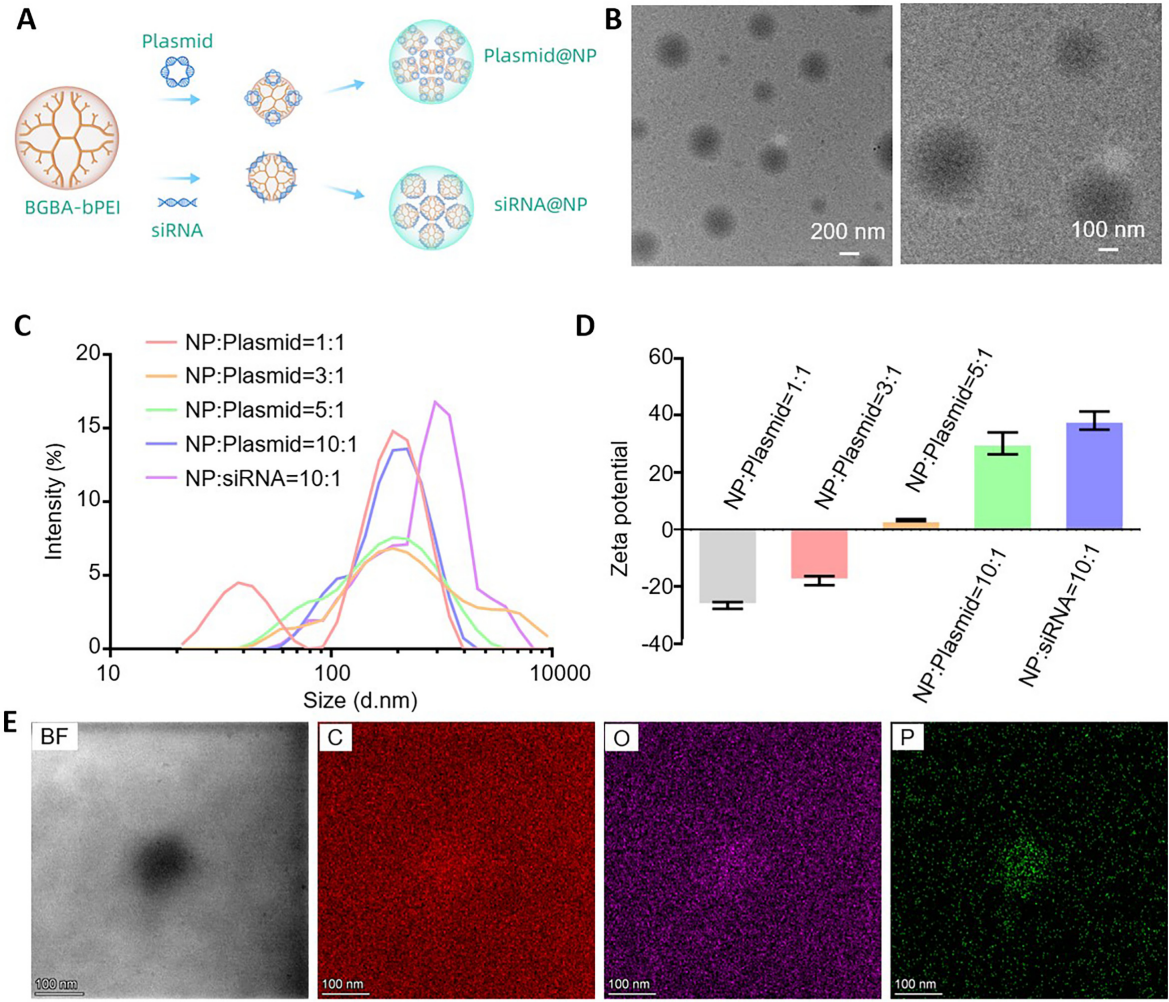
Characterization of gene-loaded nanoparticles. (a) Schematic diagram of the synthesis of nanoparticles loaded with plasmids and siRNA, respectively. (b) TEM image of blank nanoparticles. (c) Particle size distribution of gene-loaded nanoparticles. (d) Zeta potential values of gene-loaded nanoparticles. (e) The mapping energy spectrum of the nanoparticles.

Branched polyethylenimine (bPEI) is a recent discovery in cationic polymer gene transfection technology. bPEI has been utilized in a wide range of hosts and is characterized by its ease of use, low cytotoxicity, and high transfection efficiency.^21–23^ Herein, a biguanide-modified-4-aminobenzoic acid (BGBA)-modified bPEI (BGBA–bPEI) was designed, which could readily adsorb plasmids and siRNAs electrostatically and self-assemble to form nanoparticles, thus encapsulating genes within the nanoparticles. BGBA–bPEI is a large positively charged molecule that is uniformly dispersed in water (Fig. S1). When a negatively charged gene is added to the solution, the BGBA–bPEI molecule and the gene are attracted to each other as a result of their dissimilar charges. Finally, nanoparticles loaded with the gene of interest were obtained [Fig. 1(a)]. Based on the principle presented in the schematic diagram, nanoparticles comprising BGBA–bPEI, with siRNA as an example, were prepared and their morphology and particle size were observed using transmission electron microscopy (TEM). The resulting nanoparticle size was approximately 200 nm. The size was relatively uniform and dispersed in a liquid state, thus indicating that the process of constructing gene-loaded nanoparticles was successful [Fig. 1(b)].

To stably synthesize circRNA-3503 in the lesion, plasmids that could transcribe circRNA were constructed by purchasing a vector specifically designed for circRNA transcription [Fig. S2(a)]. The sequence of our target gene was integrated into this vector [Fig. S2(b)]. The resulting product was transferred into bacteria and plasmid DNA was isolated and sequenced from individual colonies. The correct clone was defined as having a vector that could successfully transcribe circRNA-3503 [Fig. S2(c)]. To verify that the gene sequence did not form a circle, divergent and convergent primers were designed. Sequences that successfully formed a circle indicated that they were successfully transcribed into RNA [Fig. S3(a)]. Based on our experimental results, groups A3 and B3 exhibited bright bands, indicating that RNA was readily transcribed from both sequences [Fig. S3(b)]. Finally, it was verified that the constructed plasmid could successfully transcribe circRNA-3503.

**FIG. 2.**
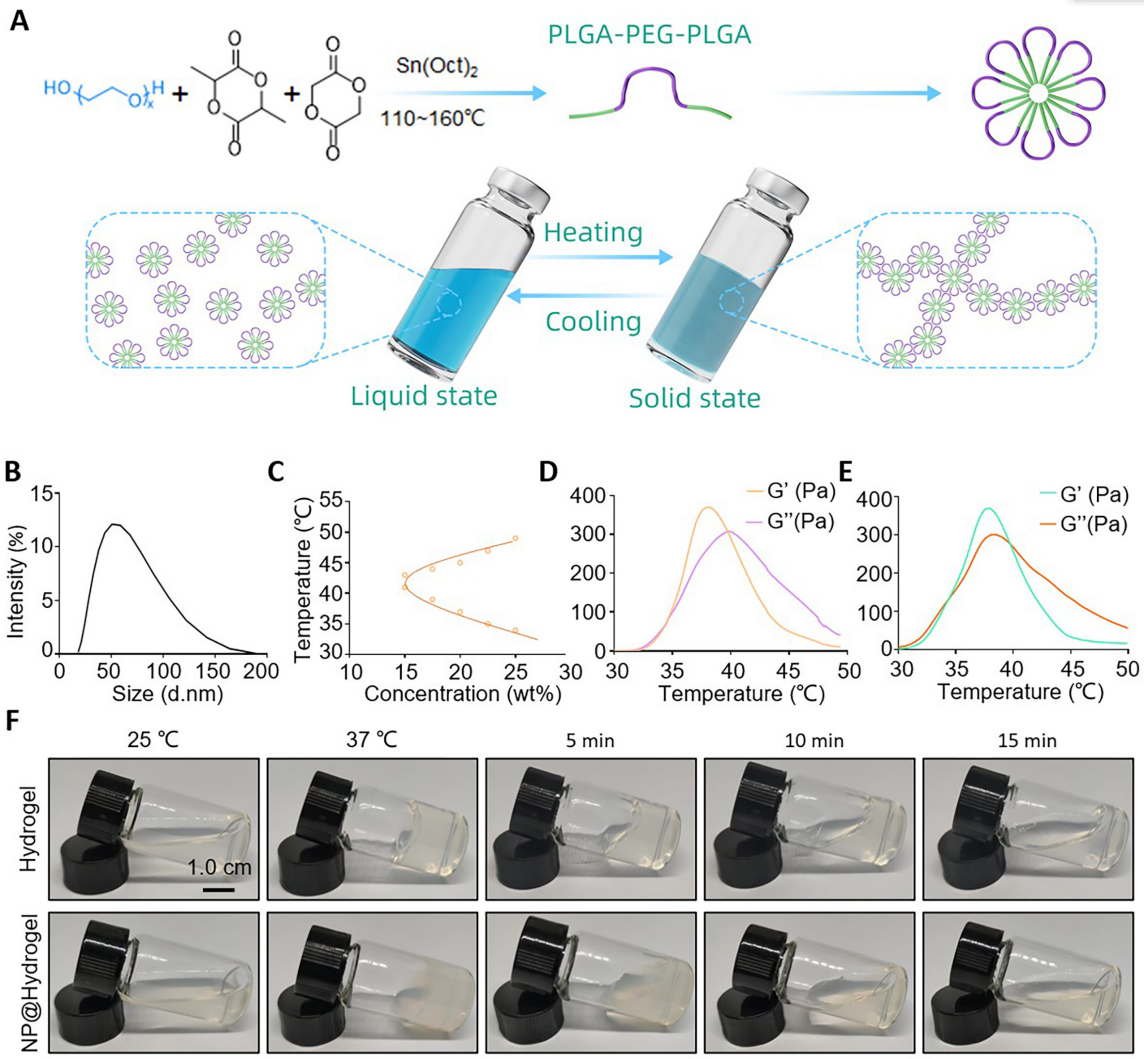
Characterization of heat-sensitive hydrogels. (a) Schematic diagram of the synthesis and mechanism of action of the heat-sensitive hydrogel. (b) Particle size distribution of nanomicelles. (c) Phase transitions in the water systems of triblock polymers. (d) Change in the modulus of the water system of triblock polymer varies with temperature. (e) Change in the modulus of the nanocomposite hydrogels varies with temperature. (f) Photographs of polymer hydrogels and nanocomposite hydrogels achieving solid and liquid transitions at different temperatures.

**FIG. 3.**
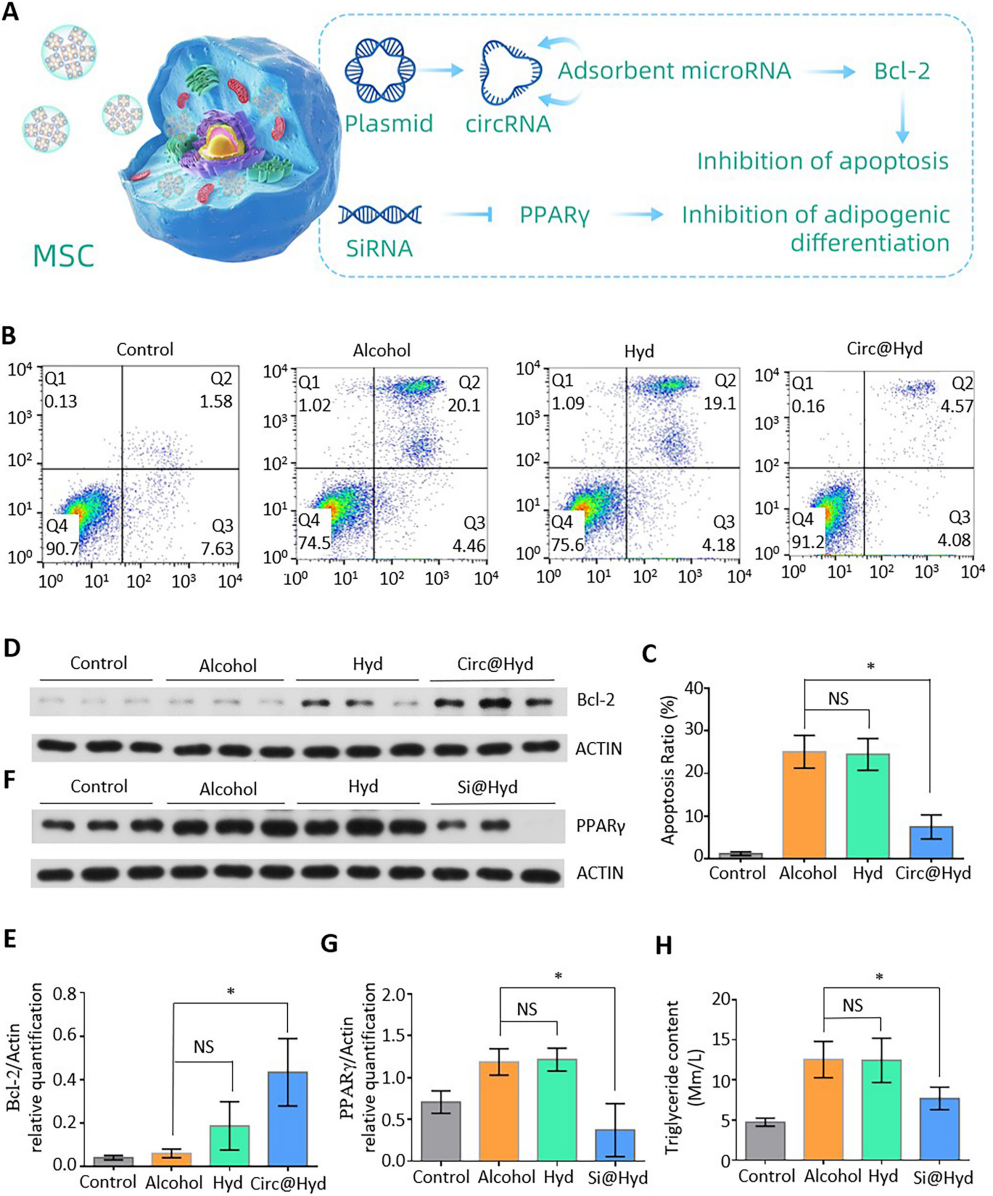
Biological effects of the nanocomposite hydrogels in the treatment of ONFH. (a) Schematic diagram of the mechanism by which nanocomposite hydrogels regulate gene expression and inhibit MSC apoptosis and adipogenic differentiation. (b) Flow cytometry was performed to detect the apoptosis of MSCs. (c) Statistical analysis of the MSC apoptosis rate. (d) Expression of Bcl-2 was detected using western blotting. (e) Quantitative analysis of Bcl-2 expression. (f) Expression of PPARγ was detected using western blotting. (g) Quantitative analysis of PPARγ expression. (h) Quantitative analysis of triglyceride content in MSCs. NS: nonsignificant; ^*^P < 0.05, ^**^P < 0.01, ^***^P <0.001.

Different proportions of BGBA–bPEI were mixed with plasmids. Using dynamic light scatterer (DLS), it was revealed that when the mass ratio of BGBA–bPEI and plasmid was 1:1, the distribution of nanoparticles was extremely wide, ranging from small to large nanoparticles, which indicated the poor stability of the composite system. When the mass ratio was increased, the nanoparticles tended to be more stable. Furthermore, when the mass ratio was increased to 10:1, the particle size was approximately 220 nm and the distribution was relatively concentrated. Nanoparticles loaded with siRNAs at a mass ratio of 10:1 were constructed, and the diameter of the resulting nanoparticles was observed to be stable at approximately 230 nm, thus demonstrating that nanoparticles loaded with genes can be successfully produced at a mass ratio of 10:1 [Fig. 1(c)]. In addition, by characterizing the surface potential, it was determined that with an increase in the BGBA–bPEI mass ratio, the surface potential of the nanoparticles gradually tended to be positive [Fig. 1(d)]. Based on the above measurements, when the mass ratio of BGBA–bPEI was relatively low, the diameter of the nanoparticles was 100–200 nm, and even a large percentage of nanoparticles were smaller than 100 nm. However, as the mass ratio increased, the diameter of the nanoparticles was concentrated around 200 nm, indicating that these nanoparticles were in a relatively stable state. Although nanoparticles with a low mass of BGBA–bPEI were small in diameter, they exhibited a negative charge, which is not conducive to uptake by cells. Therefore, nanoparticles with a mass ratio of 10:1, which were more stable, more uniform in diameter, exhibited a positive charge, and more conducive to cell uptake, were used. Next, an energy spectrum analysis of the gene-loaded nanoparticles was performed, and it was found that the interior of blank nanoparticles was rich in C and O elements. As bPEI contains phenyl boric acid, blank nanoparticles also contained the boron element. However, phosphorous (P) elements were extremely limited [Fig. S4(a)]. Subsequently, the nanoparticles loaded with plasmids and siRNA, respectively, were examined, and it was found that in addition to being rich in C, O, and B, these nanoparticles had higher amounts of P than blank nanoparticles. This P element was provided by the genes loaded on the nanoparticles [Fig. S4(b)]. Thus, nanoparticles loaded with our gene of interest were successfully constructed. To further verify that our synthesized nanoparticles were successfully loaded, a mapping energy spectrum of the nanoparticles was generated [Fig. 1(e)]. The inside of the nanoparticle was rich in P, C, and O elements, which indicated that the nanoparticles were successfully loaded.

**FIG. 4.**
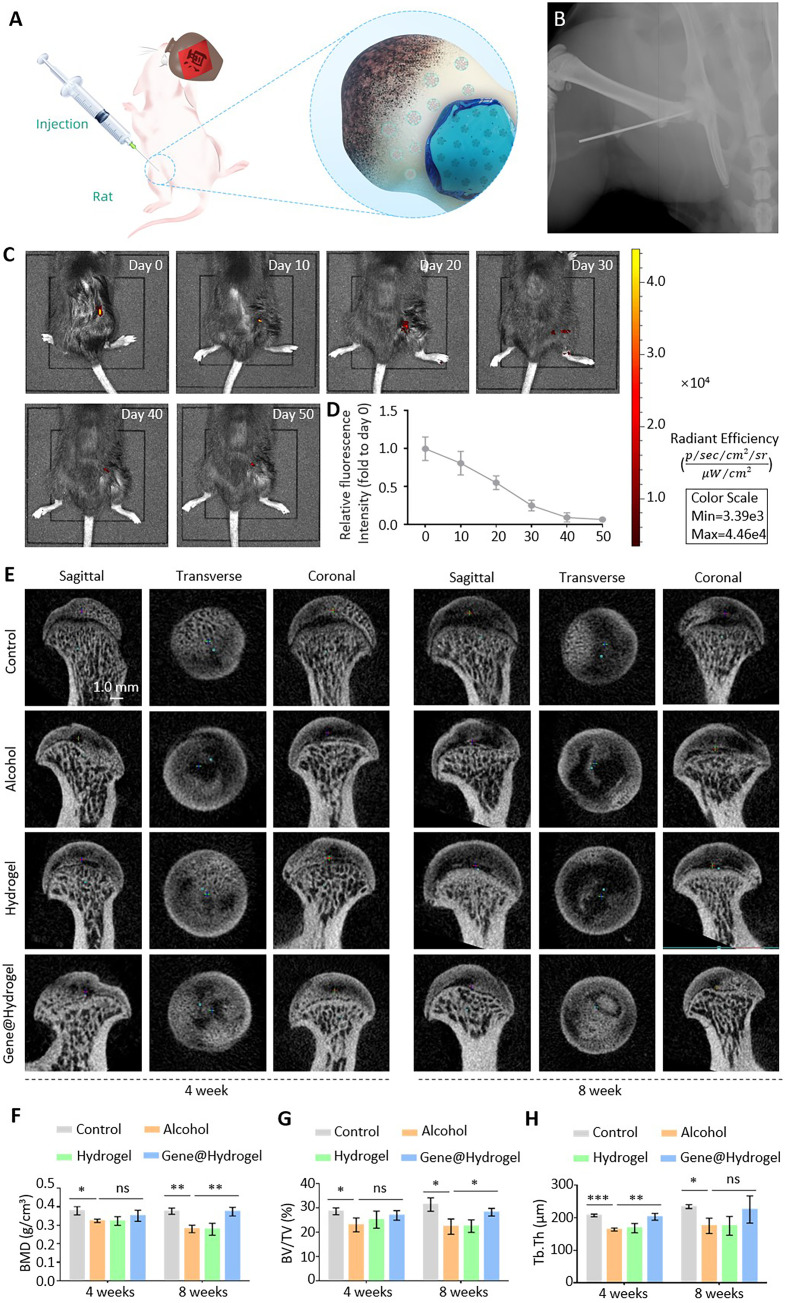
Animal experiments using the nanocomposite hydrogel for treating ONFH. (a) Schematic illustration of the generation and treatment of the rat model of alcohol-induced ONFH. (b) The liquid nanocomposite hydrogel was injected into the hip joint using a syringe. (c) Fluorescence residue test verified the indwelling of the nanocomposite hydrogel in the rats. (d) Quantitative analysis of the fluorescence residue assay. (e) Micro-CT was performed to detect femoral head necrosis in the rats. (f) Statistical analysis of BMD. (g) Statistical analysis of BV/TV. (h) Statistical analysis of Tb.Th. ns: nonsignificant; ^*^P < 0.05, ^**^P < 0.01, ^***^P < 0.001.

### Characterization of a heat-sensitive nanocomposite hydrogel

The most important characteristic of nanocomposite hydrogels is the thermal response. The appropriate concentration of hydrogel was determined using a phase transition experiment. Subsequently, rheological and macroscopic experiments were conducted to test the properties of the nanocomposite hydrogel transitioning from the liquid to solid state at 37 °C. PLGA (poly(lactic-co-glycolic acid))–PEG (polyethylene glycol)–PLGA self-assembles to form micelles in an aqueous solution, thereby establishing a hydrogel system when combined with water. At room temperature, the micelles were randomly distributed and moved freely, indicating the liquid state of the hydrogel. Upon heat-induced stimulation, the micelles exhibited a regular linear arrangement and formed an interlaced micelle network. Finally, the hydrogel solidified [Fig. 2(a)]. Based on the principle described in the schematic diagram, PLGA–PEG–PLGA triblock copolymers with a D,L-lactide (LA) to glycolide (GA) ratio of 3.6 were prepared (as determined using ^1^HNMR), and the molecular weight of PLGA–PEG–PLGA was 1740–1500–1740 kDa (Fig. S5). The amphiphilic PLGA–PEG–PLGA triblock polymer self-assembled to form nanomicelles in water, with an average particle size of approximately 40 nm [Fig. 2(b)]. The triblock polymer was tested at various concentrations (25, 22.5, 20, 17.5, and 15 wt. %) to determine the changes in the state of the polymer water system at increased temperature. Figure 2(c) shows that the sol–gel transition temperature of the polymer water system decreased with an increase in polymer concentration. When the polymer concentration was 25 wt. %, the phase transition temperature reached 33 °C and a gel was formed through *in vivo* injection. We further verified the phase transition of the polymer water system at a concentration of 25 wt. % using rheological experiments and determined its rheological properties after adding the nanocomposite system. The results [Figs. 2(d) and 2(e)] revealed that the addition of the nanocomposite system did not affect the phase transition temperature or modulus of the whole system. To characterize the sol–gel transition of the polymer hydrogel system at a macroscopic level, we acquired a macroscopic image of the system [Fig. 2(f)]. The polymer hydrogel system was transparent and flowed easily in its “sol” state at 25 °C. When the temperature was increased to 37 °C, it transiently transformed into a nonflowing, semisolid gel. After returning to room temperature, the gel gradually returned to a flowing sol state. Subsequently, we mixed the polymer hydrogel [Fig. S6(a)] and nanoparticle system [Fig. S6(b)] according to the designed mass ratio and the nanocomposite hydrogel was prepared [Fig. S6(c)]. The gel–solution transition experiment was repeated. The results indicated that the nanocomposite hydrogel system retained good heat-sensitive properties and underwent solidification during heat stimulation. These findings indicate that the gels formed using this system are reversible and meet the application requirements of various disease models.

**FIG. 5.**
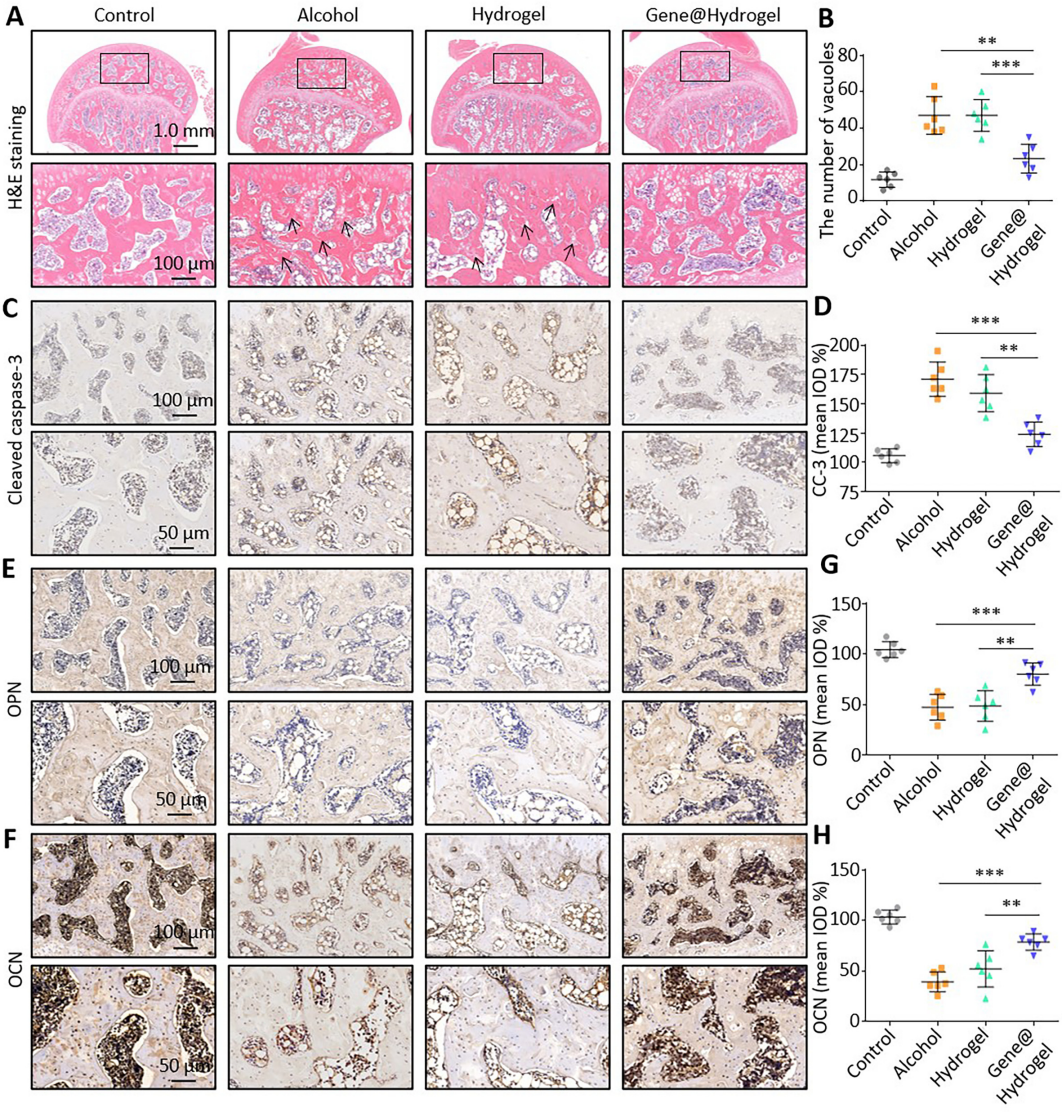
Section staining of the rat femoral head. (a) Hematoxylin and eosin staining of the rat femoral head. (b) Quantitative analysis of the number of vacuoles in the femoral head. (c) Immunohistochemical staining for cleaved caspase-3 in the femoral head. (d) Quantification of cleaved caspase-3 expression. (e) Immunohistochemical staining for OPN in the femoral head. (f) Immunohistochemical staining for OCN in the femoral head. (g) Quantification of OPN expression. (h) Quantification of OCN expression. ^*^P < 0.05, ^**^P < 0.01, ^***^P < 0.001.

### Biocompatibility of the nanocomposite hydrogel

The cytotoxicity and biocompatibility of biological materials should always be tested. We measured cell proliferation using the Cell Counting Kit-8 (CCK-8) assay, which indirectly reflects the cytotoxicity of biomaterials. We also used live/dead cell staining technology to stain and visually observe and count the dead cells. The cytotoxicity of the nanoparticles and nanocomposite hydrogels was examined. To mimic the application of the hydrogel, MSCs were selected as experimental cells and various concentrations of the biomaterials in MSC cultures were examined. The concentrations of the nanoparticles ranged from 10 to 1000 *μ*g/ml, whereas the nanocomposite hydrogel ranged from 50 to 1600 *μ*g/ml. After culturing for 48 h, the activity of the MSCs was measured using the CCK-8 assay. Using a series of MSC cultures treated with nanoparticles, the OD values of the experimental and control groups were not significantly different. Therefore, the nanoparticles exhibited no apparent cytotoxicity at the tested concentrations [Fig. S7(a)]. Furthermore, in a series of MSC cultures exposed to nanocomposite hydrogels, the OD values of the experimental and control groups were also similar [Fig. S7(b)]. Therefore, the nanocomposite hydrogels exhibited good biocompatibility and can be safely injected *in vivo*.

### Biological effects of nanocomposite hydrogels for the treatment of ONFH

The nanocomposite hydrogels were designed to regulate Bcl-2 and PPARγ in order to inhibit apoptosis and adipogenic differentiation of stem cells, respectively. Western blot analysis was used to assess the expression of the two target proteins in stem cells following exposure to the nanocomposite hydrogel. Flow cytometry was used to detect apoptosis associated with the nanocomposite hydrogel. The inhibitory effect of the nanocomposite hydrogel on adipogenic differentiation of stem cells was examined using a triglyceride kit.

We examined the underlying mechanism for the effects of the nanocomposite hydrogels for the treatment of ONFH. Our specially designed plasmid was designed to utilize the transcription system of MSCs to transcribe circRNA-3503. This circRNA can theoretically enhance the expression of Bcl-2 and inhibit alcohol-induced apoptosis of MSCs by acting as an RNA sponge and neutralizing micRNA-181c-3p. The siRNA-containing nanoparticles inhibit the expression of PPARγ in MSCs through the release of siRNA, thereby inhibiting the alcohol-induced adipogenic differentiation of MSCs. We confirmed that the normal physiological function of the MSCs was restored [Fig. 3(a)]. We added ethanol (0.09 mol/l) to the medium of the MSCs to simulate the ethanol-containing microenvironment that occurs in patients with ONFH. We used different biomaterials at various concentrations during coculture with the MSCs and verified the mechanism of MSC regulation following exposure to nanocomposite hydrogels. First, to identify the mechanism through which plasmid-loaded nanocomposite hydrogels inhibit MSC apoptosis during exposure to ethanol, we established four groups. Untreated MSCs were used as the control group, MSCs exposed to ethanol were designated the alcohol group, MSCs exposed to ethanol and blank nanocomposite hydrogels were designated the hydrogel group, and MSCs exposed to ethanol and plasmid-loaded nanocomposite hydrogels were designated the Circ@hydrogel group. To measure the inhibition of alcohol-induced apoptosis of MSCs by the nanocomposite hydrogels, flow cytometry was used [Fig. 3(b)]. The apoptosis rate of the Alcohol group was significantly higher compared with that of the control group, which indicates that long-term drinking results in apoptosis of human stem cells. However, there was no significant difference in the apoptosis rate of the alcohol-combined hydrogel group, indicating that the influence of irrelevant variables could be excluded. The apoptosis rate of the Circ@hydrogel group was significantly lower compared with that of the alcohol group. The plasmid-loaded nanocomposite hydrogel significantly inhibited alcohol-induced apoptosis of the MSCs [Fig. 3(c)]. To verify the mechanism of plasmid-induced inhibition of MSC apoptosis, we assessed the expression of Bcl-2 in the MSCs by western blot analysis [Fig. 3(d)]. Quantitative analysis of the results revealed that under regulation by the plasmid, Bcl-2 levels in the Circ@hydrogel group were significantly higher compared with that in the alcohol group [Fig. 3(e)]. Therefore, the nanocomposite hydrogel loaded with plasmids increased Bcl-2 levels in the MSCs, thus inhibiting apoptosis.

To verify whether the nanocomposite hydrogels loaded with siRNA could inhibit the alcohol-induced adipogenic differentiation of MSCs, we established four experimental groups. Untreated MSCs were used as a control group, MSCs exposed to alcohol were designated the alcohol group, MSCs exposed to alcohol and blank nanocomposite hydrogel were designated the hydrogel group, and MSCs exposed to alcohol and siRNA-loaded nanocomposite hydrogel were the Si@hydrogel group. PPARγ, a key intracellular protein that promotes the adipogenic differentiation of MSCs, was assessed by western blot analysis [Fig. 3(f)]. Quantitative analysis of the western blot results indicated that the PPARγ levels in the Alcohol group were significantly higher compared with that in the control group, indicating that long-term alcohol consumption results in lipid accumulation in the femoral head, thus aggravating ONFH. No significant differences were observed between the Hydrogel and Alcohol groups. Most importantly, PPARγ levels in the Si@hydrogel group were significantly lower compared with that in the alcohol group, indicating that the nanocomposite hydrogel loaded with siRNA significantly inhibits the alcohol-induced expression of PPARγ in MSCs [Fig. 3(g)]. To further verify whether siRNA-loaded nanocomposite hydrogels inhibit the adipogenic differentiation of MSCs, we measured triglycerides in the MSCs. The triglyceride levels of the alcohol group were significantly higher compared with that in the control group, whereas the triglyceride levels of the Si@hydrogel group were significantly compared with that in the alcohol group [Fig. 3(h)]. Thus, the nanocomposite hydrogel microspheres significantly inhibit the alcohol-induced adipogenic differentiation of MSCs.

Taken together, we designed a nanocomposite hydrogel that inhibits alcohol-induced apoptosis and adipogenic differentiation of MSCs by regulating the expression of *Bcl-2* and *PPARγ*.

### Treatment of ONFH using nanocomposite hydrogels *in vivo*

To further validate the efficacy of the nanocomposite hydrogels for the treatment of alcohol-induced ONFH, animal experiments were performed. The effective duration of nanocomposite hydrogels in the femoral head and its effect on the pathological changes of the femoral head were evaluated. We designed a fluorescence residue assay to detect the time of effect of the nanocomposite hydrogels. The bone status of femoral head was assessed by micro-computed tomography (CT). Tissue sections were prepared and immunohistochemical staining was used to observe apoptosis of cells in the femoral head and the secretion of osteoblast-related proteins. Based on the above data, we evaluated the pathology of ONFH and the progress of repair.

We generated a model of alcoholic ONFH by feeding rats with a commercially available ethanol-containing diet [Fig. 4(a)]. The rat hip joint was injected to treat ONFH [Fig. 4(b)]. The rats were acclimated to the alcohol diet for 10 days. The proportion of the alcohol diet was increased by 20% every two days up to 100% on the 10th day. The control rats were fed a control diet, and the experimental rats were fed the alcohol diet. The daily intake for each rat was limited to 100 ml. An injection needle containing a needle core was used for intra femoral head injection. This prevents the rat tissue from clogging the needle. We inserted the needle approximately 1 mm below the trochanter of the femur. The angle between the needle and the femoral shaft was approximately 120° and the insertion depth was approximately 1 cm. We verified that the needle tip reached the femoral head by x-ray. The needle core was removed and the hydrogel was injected.

To verify the degradation rate and retention time of the nanocomposite hydrogels *in vivo*, we established a fluorescence residue assay. The hydrogel was labeled with a Cy-5.5 fluorescent dye and injected into the hip joint of the rats, and the residual dose of the fluorescent dye was detected every 10 days [Fig. 4(c)]. The results indicated that the fluorescence value in the rats was approximately 55.0% of the initial value on day 20. On day 40, the fluorescence value in the rats was approximately 9.3% of the initial value, indicating that the nanocomposite hydrogel can release nanoparticles in rats for approximately 40 days [Fig. 4(d)]. Therefore, our nanocomposite hydrogel was stable in rats and achieved long-term activity.

After treatment of alcoholic ONFH, micro-CT was performed [Fig. 4(e)]. The micro-CT images revealed that the tissue in the medullary cavity of the rat femoral head in the control group was dense and uniform, whereas the alcohol and hydrogel groups exhibited large areas of dark necrotic tissue in the femoral head. Areas of necrotic tissue in the rats by the eighth week were significantly larger compared with that in the fourth week. ONFH was significantly improved in the treated Gene@hydrogel group compared the alcohol and hydrogel groups. Subsequently, bone mineral density (BMD) [Fig. 4(f)], bone volume (BV)/trabecular volume (TV) [Fig. 4(g)], and Tb.Th [Fig. 4(h)] were assessed by micro-CT. A statistical analysis revealed that BMD, BV/TV, and Tb.Th of the control group were significantly higher compared with that of the alcohol group in the fourth and eighth weeks. The results indicate that alcohol destroys the bone of the femoral head in rats and induces ONFH. The Tb.Th of the Gene@hydrogel group was significantly higher compared with that of the alcohol group in the fourth week. By the eighth week, BMD and BV/TV of the Gene@hydrogel group were significantly higher compared with that of the alcohol group. Therefore, following administration of the nanocomposite hydrogel, the bone destruction and necrosis in the femoral head were effectively inhibited. However, no significant differences between the experimental groups were observe in several cases, which may be the result of small fluctuations in physiologically relevant values or an inadequate number of samples. We plan to conduct future experiments by increasing the number of samples.

Finally, we tested the effect of the nanocomposite hydrogel on bone repair in the necrotic femoral head area. After treatment, the femoral heads of the rats were harvested for tissue sectioning, Hematoxylin and eosin (H&E) staining, and immunohistochemical staining. We defined the pathological manifestations of alcoholic ONFH as the presence of diffuse granular vacuolar cells in the trabecular bone with pyknotic nuclei and necrosis in the surrounding bone marrow.^24–26^ H&E staining [Fig. 5(a)] revealed no histopathological changes associated with ONFH in the control group; however, in the alcohol group, diffuse vacuolar areas (black arrows) were observed in the trabecular bone of the femoral head and a large amount of necrotic cell debris had accumulated in the medullary cavity. This indicated that the alcohol-induced ONFH rat model was successfully established. Meanwhile, a significant reduction in osteonecrosis was observed in the Gene@hydrogel group, indicating that the nanocomposite hydrogel had a significant effect on ONFH. To further analyze the histopathological results, the vacuoles in the trabecular bone were analyzed [Fig. 5(b)]. The alcohol group had significantly more vacuoles compared with the control group; however, there was no significant difference in the number of vacuoles between the alcohol and hydrogel groups, and thus, irrelevant variables were excluded by comparing the two groups. Most importantly, the number of vacuoles in the Gene@hydrogel group was significantly lower compared with that in the alcohol group, indicating that the nanocomposite hydrogel significantly inhibits disease progression.

To detect apoptosis in the necrotic tissue of the femoral head, we performed a cleaved caspase-3 immunohistochemical staining of rat femoral head sections [Fig. 5(c)]. In the alcohol group, significant positive staining was observed in the trabecular bone, whereas positive staining was significantly reduced in the Gene@hydrogel group. Furthermore, a semiquantitative statistical analyses of the immunohistochemical staining results was performed [Fig. 5(d)]. The levels of cleaved caspase-3 in the Alcohol group were significantly higher compared with that in the control group; however, no significant differences were observed in comparison with the hydrogel group. Meanwhile, the cleaved caspase-3 levels in the Gene@hydrogel group were significantly lower compared with that in the alcohol group. These findings suggest that the nanocomposite hydrogel effectively inhibit the alcohol-induced MSC apoptosis.

Osteopontin (OPN) and osteocalcin (OCN) are osteogenic markers expressed in bone marrow MSCs during osteogenic differentiation and calcium mineralization. We performed immunohistochemical staining for OPN [Fig. 5(e)] and OCN [Fig. 5(f)] to explore the osteogenic activity in the rat femoral head. Figures 5(g) and 5(h) show the results of the statistical analysis of the immunohistochemical staining results of OPN and OCN, respectively. The OPN and OCN levels in the alcohol group were significantly lower compared with that in the control group. These results indicate that exposure to alcohol significantly inhibits the formation of new bone in the femoral head, thereby further aggravating ONFH. However, the levels of OPN and OCN in the Gene@hydrogel group were significantly higher compared with that in the alcohol group. These results suggest that the nanocomposite hydrogel can significantly improve osteogenic activity in the femoral head of alcohol-induced ONFH rats, thus promoting the repair of ONFH-associated lesions. In conclusion, our findings demonstrate that the nanocomposite hydrogels can significantly inhibit alcohol-induced apoptosis of MSCs, promote osteogenic activity in the femoral head, and enhance the repair of necrotic sites during treatment of alcohol-induced ONFH.

## CONCLUSION

Osteonecrosis^27^ and bone regeneration^28^ were the focus of this study. Presently, much progress has been made using functional biological materials for the treatment of osteonecrosis,^29^ of which nanocomposite hydrogels show great potential.^30^ In this study, we created a novel, heat-sensitive nanocomposite hydrogel system with a secondary nanostructure that regulates gene expression to achieve long-term gene regulation of focal cells. The nanocomposite hydrogel is in a liquid state at room temperature and is injectable. When injected into the body of patients with ONFH suffering from long-term alcoholism, the hydrogel undergoes solidification during heat stimulation induced by body heat, thereby gaining the ability to be retained in the body for a prolonged time period. With the gradual degradation of the nanocomposite hydrogel *in vivo*, the internal secondary nanostructures can be released continuously. Nanoparticles can carry plasmids and siRNA into the lesion stem cells, thus promoting the expression of Bcl-2 and inhibiting stem cell apoptosis, secretion of PPARγ, and adipogenic differentiation. Finally, the physiological activity of stem cells around the necrotic femoral head area can be restored and ONFH repair promoted. Our *in vivo* experiments demonstrated that the nanocomposite hydrogel can be indwelled *in vivo* for a long time to achieve a long-term treatment effect by promoting bone reconstruction in the femoral head and ameliorating alcohol-induced ONFH. Moreover, the nanocomposite hydrogels provide a novel treatment option for alcohol-related diseases and have the potential to be used for other gene therapy applications.

## METHODS

### Materials

Polyethylene glycol (PEG)-1500 was obtained from Aladdin Biochemical Technology Co., Ltd. (Shanghai, China). Glycolide (GA) and D,L-lactide (LA) were obtained from Ming Zhong Biotechnology Co., Ltd. (Hangzhou, China). Stannous octoate [Sn(Oct)_2_], branched polyethylenimine (bPEI; 25 kDa), and 4-aminobenzoic acid (GBA) were purchased from Sigma-Aldrich. Plasmids and siRNA were obtained from Jiman Biotechnology Co., Ltd. (Shanghai, China). All other reagents are described in detail in the text.

### Synthesis and characterization of nanoparticles

GBA (4 mmol) and hydrochloric acid (4 mmol) were diluted in 20 ml of de-ionized water, followed by treatment with dicyandiamide (8 mmol), and incubated at 80 °C for 6 h in the dark. Then, the zoic acid of biguanide-modified GBA (BGBA) was obtained via sedimentation in acetone. To synthesize BGBA-modified bPEI, BGBA, dicyclohexylcarbodiimide, and N-hydroxysuccinimide (at a molar ratio of 1:1.3:1.2) were dissolved in 20 ml of dimethyl formamide (DMF) solution and incubated for 6 h. Subsequently, bPEI (1.2 *μ*mol) dissolved in dimethyl sulfoxide (DMSO) was added followed by 2 ml of triethylamine. The mixture was stirred for 7 days at 25 °C. DMSO in the solvent was removed by dialysis and BGBA-modified bPEI was obtained through freeze-drying.

The resulting BGBA-modified bPEI was dissolved in water and an equal volume of plasmid was added to the mixture to produce the following mass ratios of bPEI to plasmid: 1:1, 3:1, 5:1, and 10:1. The mixtures were maintained for 30 min in 25 °C to obtain the composite nanosystem. Using the same method, we prepared nanoparticles with a mass ratio of 10:1.

### Synthesis and characterization of the heat-sensitive nanocomposite hydrogel

From our previous study,^31^ we found that PEG, PLGA–PEG–PLGA triblock copolymers can be synthesized by ring-opening copolymerization of LA and GA under the catalytic activity of Sn(Oct)_2_. The specific steps in the synthesis were as follows. First, PEG (0.01 mol) was used to remove water, and LA and GA were added without oxygen. Subsequently, the remaining water was removed at 80 °C. After melting all of the monomers, Sn(Oct)_2_ (0.2 wt. % monomer) was added and the mixture was stirred at 150 °C for 12 h under argon protection. At the end of the reaction, the product was added to water at 80 °C, washed three times, and the PLGA–PEG–PLGA powder was obtained by lyophilization. Finally, 4% PLGA–PEG–PLGA and 2.5% nanoparticles were dissolved in water and stirred for 4 h at 25 °C to obtain the nanocomposite hydrogels.

### Biomaterial characterization

The molecular weight of the synthesized PLGA–PEG–PLGA triblock polymer and molar ratio of the monomer were characterized using proton nuclear magnetic resonance (^1^H NMR). DLS was used to characterize the particle size of the PLGA–PEG–PLGA triblock polymer self-assembly. The temperature-sensitive characteristics and phase transition temperature of the water system of the triple block polymer were determined using an inverted tube and rheology.

Particle size analysis and determination of the surface potential change of the nanoparticles were done by DLS to verify the stability of the composite nanoparticles and law of surface potential changes. The morphology and particle size of the composite nanoparticles were confirmed using TEM.

### Cytotoxicity assay

The CCK-8 assay kit was used to detect the cytotoxicity of the biomaterials. MSCs were cultured in 48-well plates for 24 h. Subsequently, various concentrations of nanoparticles and nanocomposite hydrogels were added to each well. The cells were cultured in an incubator for 48 h. Next, the cells were incubated for 30 min in the dark after adding the developing reagents, according to the manufacturer's instructions. The liquid from each well was aspirated and transferred into a 96-well plate. The 96-well plate was placed under a fluorescence microscope (PCOM, Nikon, Japan) and the absorbance was recorded.

### Flow cytometry

MSCs were processed according to the experimental protocol and were collected by digestion and centrifugation (as per the centrifugation conditions of cells collected during normal passaging). After washing twice with phosphate-buffered saline, the MSCs were resuspended in 500 *μ*l of 1× binding buffer. Subsequently, 5 *μ*l of annexin V-(fluorescein isothiocyanate) FITC and 10 *μ*l of propidium iodide dye were added to each tube, and the contents were mixed well. After incubating in the dark for 5 min at room temperature, flow cytometry was performed to detect apoptosis. The results showed a diagram that mainly divided MSCs into three states: surviving cells, early apoptotic cells, and late apoptotic cells.

### Western blot analysis

MSCs cell extracts were prepared by a standard protocol and the protein concentration was determined. Protein markers and samples were separated by sodium dodecyl sulfate (SDS) polyacrylamide gel electrophoresis at 80 V for 30 min (until the protein samples and markers entered the lower separating gel and multiple marker bands appeared). The voltage was then increased to 120 V, and electrophoresis continued until the bromophenol blue extended outside the rubber strip. Subsequently, the protein bands were transferred to poly(vinylidenefluoride) (PVDF) membranes, blocked, and incubated overnight at 4 °C in a buffer containing primary antibodies. Subsequently, the samples were incubated with secondary antibodies (2 h, 25 °C) and developed. The data were recorded using the instrument.

### Establishment of an animal model

The Lieber–Decarli standard alcohol liquid model diet was used to establish a rat model of alcohol-induced ONFH. Alcohol liquid feed (No. TP 4030A) and control liquid feed (No. TP4030C) were purchased from Trophic Animal Feed High-tech Co. Ltd (Nantong, China). One week before initiating the experiment, the rats were fed with different proportions of the alcohol liquid model diet (20%–100%) after 24 h of fasting, enabling acclimatization to the alcohol diet. After the initiation of the experiment, the dietary intake of each group was strictly limited to the lowest amount that any group had consumed the day before to minimize differences with respect to food intake.

### Micro-computed tomography (micro-CT)

After fixing the rat samples, a high-resolution micro-CT (Sky Scan 1172, Belgium) was used to detect ONFH. The scan spacing was set to 9 *μ*m, and the images were acquired at 35 kV and 220 mA. The trabecular bone volume fraction (BV/TV), trabecular bone thickness (Tb.Th), and BMD were calculated using CT Vol software.

### Morphology and immunohistochemical staining

Samples were collected and placed in a decalcification solution containing 10% EDTA for one month while shaking, and the decalcification solution was replaced daily. After decalcification, the bone tissue samples underwent gradient dehydration, they were embedded in paraffin, and cut into 5-mm thick sections in a coronal plane. H&E staining was done to evaluate the overall morphology of the femoral head. Moreover, immunohistochemical staining for osteocalcin (OCN) and osteopontin (OPN) was performed to determine the change in osteogenic ability in the rat femoral heads.

### Statistical analysis

All data are presented as the mean ± standard deviation. A comparison between two groups was performed using a Student's t-test. A P value of <0.05 was considered statistically significant.

## SUPPLEMENTARY MATERIAL

See the supplementary material for additional biomaterial characterization data and cytotoxicity data.

## Data Availability

The data that support the findings of this study are available from the corresponding authors upon reasonable request.
